# Autoimmune Thyroid Disease and Psoriasis Vulgaris after COVID-19 in a Male Teenager

**DOI:** 10.1155/2021/7584729

**Published:** 2021-07-27

**Authors:** Nadia K. Qureshi, Sanjay K. Bansal

**Affiliations:** Department of Pediatrics, Loyola University Medical Center, Maywood, IL, USA

## Abstract

COVID-19 is implicated in triggering autoimmune, dermatologic, and thyroid diseases. We present a first known case of development of Graves' disease and psoriasis vulgaris in a previously healthy male teenager without any family history, diagnosed after COVID-19 infection. Evaluation of “long COVID syndrome” should include thorough history and thyroid evaluation.

## 1. Introduction

Since the emergence of severe acute respiratory syndrome coronavirus 2 (SARS-CoV-2) in December 2019, a plethora of variable clinical presentations and postviral short-term and long-terms effects on multiple organs have been implicated.

SARS-CoV-2 utilizes the angiotensin-converting enzyme 2 (ACE-2) as a cellular entry receptor [1]. As ACE-2 is known to be expressed by several tissues, it has been hypothesized that SARS-CoV-2 would infect not only the respiratory tract cells but also other tissue cells expressing ACE-2. Recently, a study demonstrated that the mRNA encoding for the ACE-2 receptor is expressed in thyroid follicular cells [2]. There have been only a few cases of subacute thyroiditis as well as Graves' disease associated with coronavirus disease 2019 (COVID-19) reported in literature studies [3–6]. Graves' disease (GD) is an autoimmune disease of the thyroid typically accompanied by an enlarged thyroid gland and occasionally ocular manifestations. The excess stimulation of thyroid-stimulating hormone (TSH) receptors by thyroid receptor antibodies generates an unregulated production and secretion of thyroid hormone, resulting in thyrotoxicosis. Genetic predisposition and epigenetic modulation play an important role in the pathogenesis of GD along with environmental factors such as medications, stress, smoking, and certain viral infections [7]. Psoriasis is also an autoimmune chronic inflammatory skin disease that can be triggered by trauma, infections, drugs, sunlight, stress, alcohol, smoking, and HIV. Guttate psoriasis is classically associated with streptococcal infections though viral infections have been implicated as well. In a few COVID-19 patients, atopic dermatitis and psoriasis have been aggravated as preexisting skin diseases [8].

To our knowledge, this is the first case of development of autoimmune thyroid disease and psoriasis vulgaris in a healthy male adolescent after a mild case of COVID-19 infection.

## 2. Case

A 13-year-old previously healthy male presented 8 weeks after his initial diagnosis of COVID-19 infection to his pediatrician with complaints of dizziness, fatigue, difficulty sleeping, and a presyncopal episode. The patient was unable to participate in daily activities without getting easily fatigued. Parents reported increased appetite but still a documented weight loss of 8 lbs (5.3% bodyweight) since his COVID-19 illness over a period of 2 months. Further review of system revealed significant heat intolerance requiring multiple fans pointed at him while sleeping without any covers. He also had an itchy groin rash for which he was using topical cotrimoxazole cream with some improvement. He denied any sexual activity or illicit drug use. He was diagnosed with COVID-19 on a nasal PCR test 8 weeks prior to current presentation. He had a mild course with low-grade fever, congestion, cough, and body aches that resolved in few days. All family members were positive at the same time with his father requiring a three-week hospital stay for COVID-19 pneumonia. Other than his recent COVID-19 illness, he had no significant past medical history. He was born in Mexico and had immigrated to the United States three years ago. His immunizations were up to date. Family history was negative for any rheumatological or autoimmune disorders. He was at 98th percentile for height and 93rd percentile for weight with a BMI of 81st percentile for age. Examination was significant for tachycardia at 102 beats per minute with mild exophthalmos and palpable thyroid. He had an erythematous lesion in intertriginous left groin. His electrocardiogram showed sinus tachycardia. Results of initial blood work revealed elevated free T4 at 2.5 ng/dL (normal range: 0.7–1.5 NG/DL), undetectable TSH at <0.01 uIU/mL (normal range 0.50–4.80 uIU/mL), normal complete blood count, normal sedimentation rate, and a negative QuantiFERON TB gold test. Further workup showed elevated antithyroid peroxidase (TPO) antibodies at 946.2 IU/ml (normal <9.0 IU/ML) and elevated thyroid-stimulating immunoglobulins (TSIs) at 28.2 IU/L (normal <0.10 IU/L). Given the presence of antibodies for both Graves and Hashimoto's (with TSI predominance), he was diagnosed with hyperthyroidism from autoimmune thyroid disease. He was started on methimazole 10 mg once daily at that time. At 2-month follow-up, he had some symptomatic improvement in his energy levels with some weight gain, but still had palpitations and difficulty sleeping with some heat intolerance. Propranolol 10 mg twice daily was added for symptomatic relief. He also reported worsening of his groin rash despite changing to different antifungal creams. Upon dermatology consultation, he was determined to have psoriasis vulgaris and was treated with topical steroids resulting in complete resolution.

At 4-month follow-up, he showed biochemical improvement while on thionamide therapy, as his free T4 had normalized to 1.2 ng/dl, but his TSH remained at < 0.01 uIU/ml. However, he clinically continued to have mixed thyroid symptomology with fatigue, weight gain, poor concentration, insomnia, depression/anxiety, heat intolerance, and palpitations. This reiterates that long COVID-19 may persist in some long haulers even after other medical conditions are controlled.

## 3. Discussion

SARS-CoV-2 infection is increasingly being recognized as a trigger for rheumatological and autoimmune disorders by various mechanisms including molecular mimicry, bystander killing, and epitope spreading [9]. COVID-19 shares many of the same features as with autoimmune diseases in terms of pathogenesis, immune responses, and some clinical manifestations. Robust immune response resulting in autoantibodies occur in both COVID-19 patients and autoimmune disorders [9, 10]. As with any autoimmune disorder, both genetic predisposition and environmental trigger play an important role. This case highlights the development of autoimmune disorders after a COVID-19 infection in a previously healthy pediatric patient with no apparent risk factors.

There is suggestive evidence of molecular mimicry that comes from thyroid cell expression of HLA class II molecules in patients with autoimmune thyroid disease (Graves' disease) that are not expressed in normal thyroid tissue, where MHC (HLA-DR) expression is theorized to be a direct result of viral infection or induced by T-cell cytokines (IFN-gamma and IFN-alpha) [10, 11]. These antigen-presenting cells then may initiate autoimmune response leading to antibody production to TSH receptor (TSI).

Various skin manifestations have been reported in the weeks following COVID-19 infection. Skin rashes can take many forms including vesicular, maculopapular, urticarial, or chilblain-like lesions on the extremities (so-called COVID toe) [12]. Psoriasis vulgaris has not been described as one of them. Psoriasis vulgaris can be a great mimicker of tinea cruris. Our patient was initially misdiagnosed with tinea cruris, but persistence of symptoms and lack of improvement with various antifungal therapies led to the actual diagnosis.

Post-acute COVID-19 (“long COVID”) seems to be an evolving term that signifies persistent symptoms in patients who have tested positive for SARS-CoV-2 beyond three weeks [13]. Fatigue seems to be one of the most prominent symptoms for such long haulers. Incidence in mental health illnesses in these patients is increasingly being recognized. As we learn more about SARS-CoV-2 infection and its long-term effects, we should be evaluating these patients carefully with a thorough history and examination. Further testing should be ordered selectively and for specific clinical indications. There is much overlap between symptoms of depression and many other medical conditions including thyroid that may be mistaken to be part of post-COVID-19 syndrome. Given the significant effects of SARS-CoV-2 infection on cardiovascular system including either as part of multisystem inflammatory syndrome in children (MIS-C) or other cardiac complications such as myocarditis, arrhythmias, and heart failure, there has been a lot of focus on cardiac evaluation post-COVID-19 infection in pediatric population [14–16] but not much information on thyroid evaluation. Thyroid evaluation should be strongly considered in patients presenting with fatigue, dizziness, and weight loss.

## Data Availability

No data were used to support this study.

## Abbreviations

COVID-19: Coronavirus disease-2019
SARS-CoV-2: Severe acute respiratory syndrome coronavirus 2
Long COVID: Long-term effects of COVID-19 infection
Long haulers: Patients suffering from long-term effects of COVID-19 infection
TSH: Thyroid-stimulating hormone.

## Additional Points

*Summary.* This report describes a previously healthy adolescent male with no family history of autoimmune disorders who developed autoimmune thyroid disease and psoriasis soon after COVID-19.
